# Supplementary material for: Accentuated Peripheral Blood NK Cytotoxicity Forms an Unfavorable Background for Embryo Implantation and Gestation
Source: Diagnostics (Basel). 2022 Apr 6;12(4):908. doi: 10.3390/diagnostics12040908 (PMC9031692; doi:10.3390/diagnostics12040908)
Supplement: Supplementary file 1 [file diagnostics-12-00908-s001.zip › diagnostics-1641545-supplementary.pdf]

Supplemental Figure S1

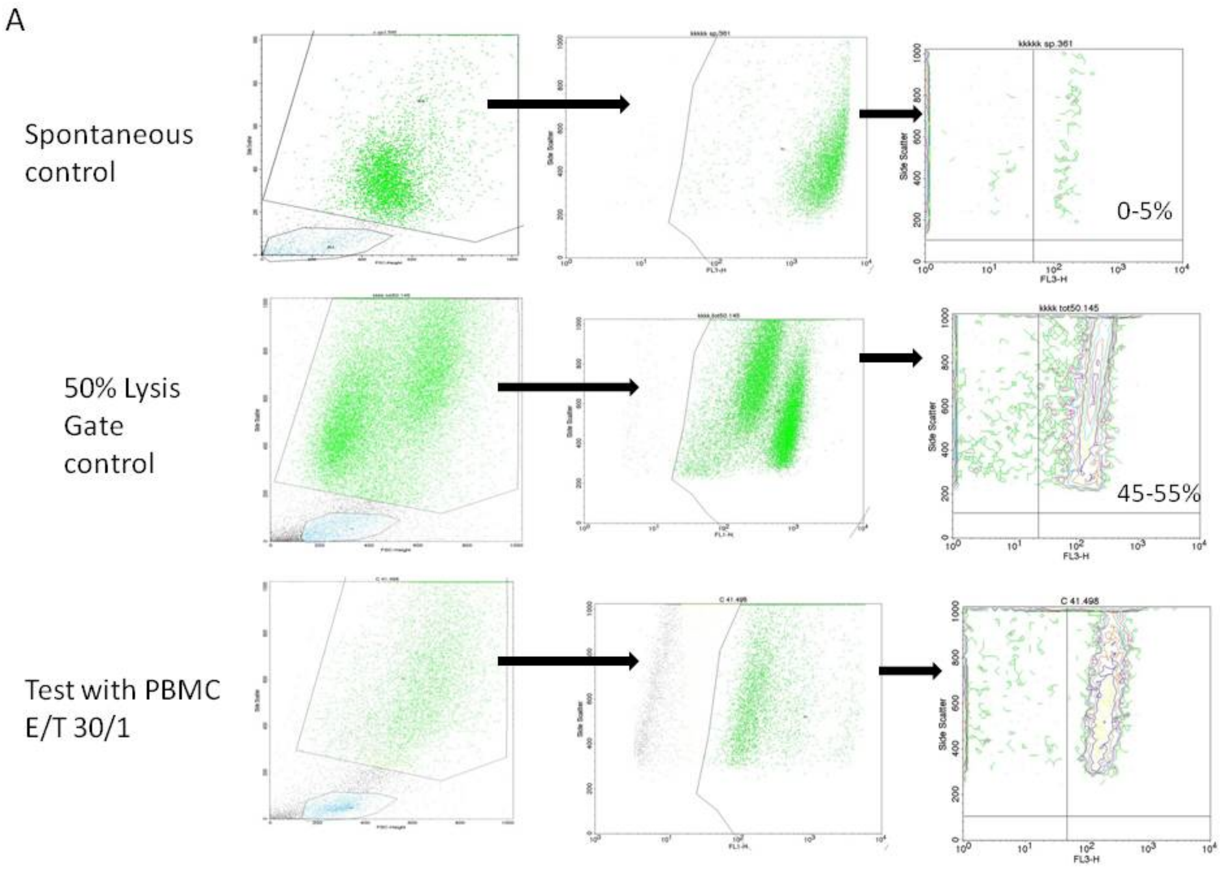

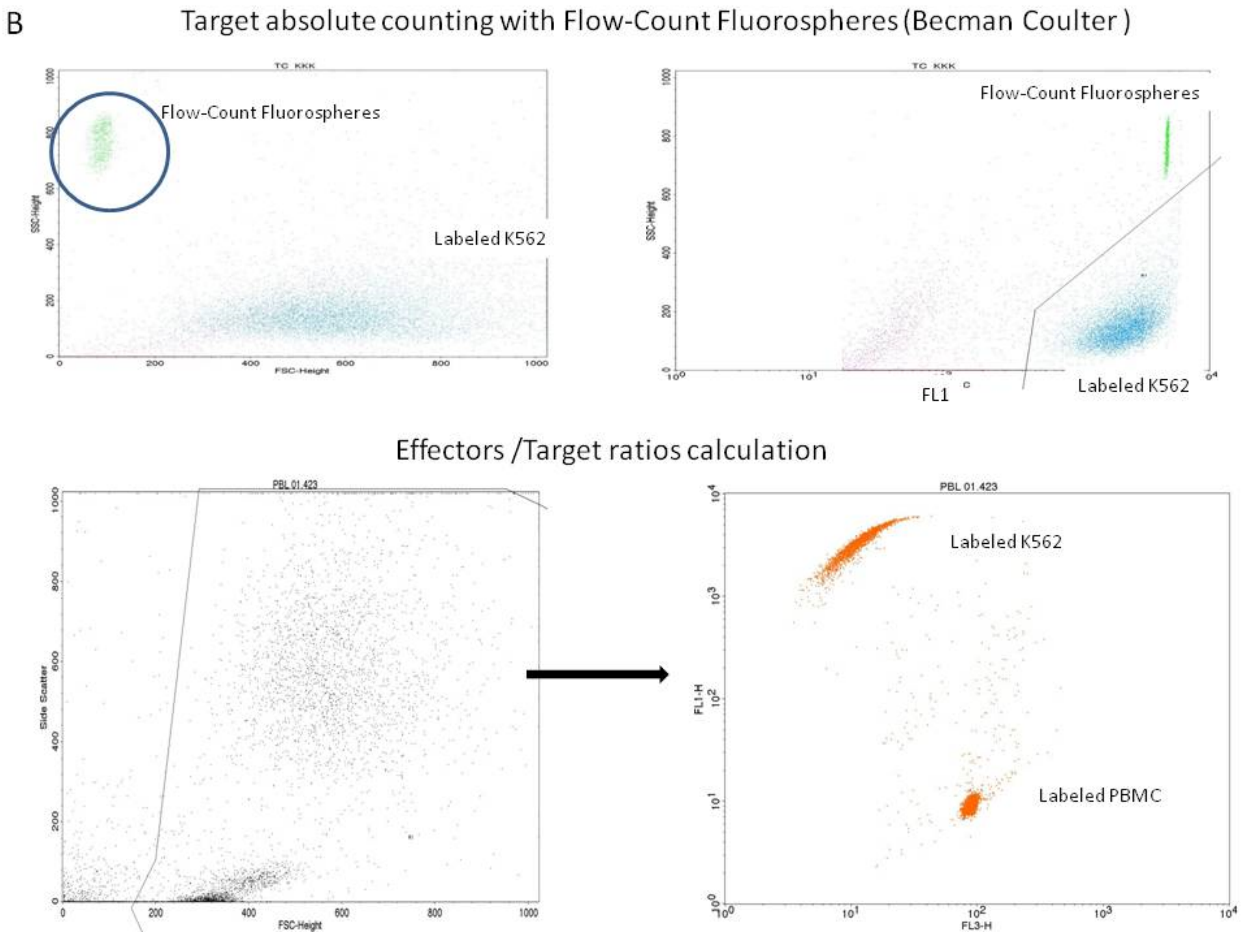

Figure S1. (**A**) Gate ant test strategy for spontaneous levels, 50% Lysis and individuals NKcytotoxic activity investigation. (**B**) Gate strategy for K562 absolute counting with Flow-Count Fluorospheres and effectors/target ratios calculation.
